# Diffusion of innovations of digital and remote services in Finnish community pharmacies during the early phase of the COVID-19 pandemic: A National Survey

**DOI:** 10.1016/j.rcsop.2026.100827

**Published:** 2026-07-25

**Authors:** Paula Timonen, Katja Ojanperä, Eeva Savela, Marja Airaksinen

**Affiliations:** a The 4th Pharmacy of Lappeenranta, Division of Pharmacology and Pharmacotherapy, Faculty of Pharmacy, University of Helsinki, Viikinkaari 5 E (PL 56), 00014, Finland; b Hospital Pharmacy, The wellbeing services county of Southwest Finland, Division of Pharmacology and Pharmacotherapy, Faculty of Pharmacy, University of Helsinki, Viikinkaari 5 E (PL 56), 00014, Finland; c Pharmacy Owner, The 10th Pharmacy of Espoo, Division of Pharmacology and Pharmacotherapy, Faculty of Pharmacy, University of Helsinki, Viikinkaari 5 E (PL 56), 00014, Finland; d Division of Pharmacology and Pharmacotherapy, Faculty of Pharmacy, University of Helsinki, Viikinkaari 5 E (PL 56), 00014, Finland

**Keywords:** Community pharmacy, Digital service, Remote service, Innovation, Diffusion of innovation, COVID-19 pandemic, Finland

## Abstract

**Aim:**

To examine Finnish community pharmacy owners' readiness to adopt innovations and its association with implementation of digital and remote services during the first wave of the COVID-19 pandemic (December 2019–October 2020).

**Methods:**

A nationwide cross-sectional survey was conducted among all private community pharmacy owners in Finland in October–November 2020. A study-specific electronic questionnaire assessed changes in digital and remote service provision. Innovation readiness was measured using a validated instrument based on Rogers' Diffusion of Innovations theory, and pharmacies were classified as early or late adopters. Data were analysed using chi-square tests, Wilcoxon signed-rank tests, and multivariable ordinal logistic regression.

**Results:**

Responses were received from 175 of 619 pharmacies (28%). Remote service provision increased significantly. Availability of medicine pick-up lockers rose from 21% to 39% (*p* < 0.001), while online pharmacy services increased from 25% to 34% (*p* < 0.001). According to Rogers' adopter categories, 43% of pharmacies were classified as early adopters. These pharmacies offered a broader range of digital services than late adopters (*p* < 0.01). Innovation readiness was higher among pharmacy owners younger than 50 years than among those aged 50–59 years (*p* = 0.009) and ≥60 years (*p* = 0.039). Pharmacies dispensing more than 100,000 prescriptions annually showed greater willingness to adopt innovations than those dispensing fewer than 40,000 (*p* = 0.016). Innovation readiness was significantly associated with remote service adoption.

**Conclusions:**

Finnish community pharmacies rapidly expanded digital and remote services in response to COVID-19. Innovation readiness was an important determinant of service development, although the low response rate may have overestimated innovativeness.

## 1 Introduction

The COVID-19 pandemic has been the most recent global disease outbreak to test the capacity of health services and the adequacy of medical interventions in responding to an emerging public health threat. The pandemic was officially declared by the World Health Organization (WHO) in March 2020.^1^ The situation was challenging worldwide because the characteristics of the virus causing the disease were still largely unknown, and evidence-based vaccines and treatments were not yet available. Health services had to be rapidly reorganized and prioritized to meet changing healthcare needs. Healthcare systems operated at the limits of their capacity to manage large numbers of infected individuals and to protect both residents and healthcare personnel from infection.

Community pharmacies became frontline healthcare providers from the beginning of the pandemic, offering highly accessible support to the public.^2^ Residents urgently needed professional advice and treatment guidance to relieve COVID-19 symptoms and prevent the spread of the disease. In many cases, other healthcare facilities were overcrowded, and community pharmacies were the only local healthcare providers that residents could access without a scheduled appointment. This sudden increase in demand for pharmacy services required community pharmacists to develop new ways of managing large customer volumes. Thus, community pharmacies played an important role in the healthcare system during the COVID-19 pandemic by ensuring the access and continuity of care while alleviating pressure on other healthcare providers.^3^

Because of the high risk of infection, community pharmacies had to find new ways of serving and interacting with their customers to minimize physical contact within pharmacy premises. Prior to the COVID-19 pandemic, several digital and remote pharmacy services were already available, including online pharmacies and video consultation technologies to support feasible medicine delivery, dispensing and counselling to ensure safe medication use.^4^, ^5^, ^6^ The COVID-19 pandemic further increased the demand for remote and digital services in this respect.^7^, ^8^, ^9^ Experiences with remote pharmacy services had primarily been gained and reported in North America and Western Europe.^10^ In Scotland, for example, video consultation technology had been used to support medicine delivery,^5^ while in Germany it had been used to identify drug-related problems.^6^ Although the COVID-19 pandemic was not considered the sole driver of change, it was reported to have played an important role in expanding community pharmacy services.^11^

## 2 Finland as a study context

Finland is a Nordic country with a population of 5.6 million.^12^ It has been a member of the European Union since 1995. The Finnish healthcare system consists of public healthcare services supplemented by private healthcare and occupational health services.^13^ The public health insurance system covers health services, including prescription medicines, for all permanent residents.

Finland's community pharmacy system is privately owned, with pharmacy owners required to be licensed pharmacists holding a pharmacy license issued by the Finnish Medicines Agency.14., 15 Community pharmacies are responsible for the supply and distribution of prescription and non-prescription medicines to the public in outpatient care, having a statutory obligation to ensure safe and rational use of medicines.^14^ At the end of 2019, there were 819 pharmacy outlets in Finland: 623 of them were main pharmacies, which operated 196 branch pharmacies in total, each main pharmacy being allowed to run maximum three branch pharmacies.^16^ The aim of the pharmacy license system is to ensure the maintenance of nationwide access to pharmacy services, supplemented by branch pharmacies and online services.

Information technology has been used in Finnish community pharmacies since the early 1980s (117). Today, electronic systems support a wide range of activities, including prescription processing, patient data management, medication counselling, medication risk assessment, patient safety reporting, logistics, procurement, reimbursement management, administration, and business planning.^15^, 17., 18, 19, 20 Digitalization has been actively promoted by the Association of Finnish Pharmacies through a series of national digital service strategies, the most recent published in 2017, with the goals of maintaining access to local pharmacy services and supporting ageing at home.^21^

Since the introduction of electronic prescribing in 2011, prescription medicines have also been available through online pharmacy services.^22^ These services, including mobile applications and other digital platforms, are strictly regulated with regard to data security, strong customer authentication, prescription verification, and the provision of appropriate medication information by community pharmacists.14., 23., 24.

The long-term development of Finnish community pharmacy practice has focused on strengthening pharmacies' role in healthcare through the promotion of rational pharmacotherapy and the use of digital solutions to improve medication management and access to medicines.

The innovation readiness of Finnish community pharmacies was examined in 2006 using Rogers' Diffusion of Innovations theory.^17^ Community pharmacy owners reported a greater readiness to adopt innovations than predicted by Rogers' general adoption model.^25^ Nevertheless, the implementation of new information technologies has progressed more slowly than expected.^17^ Despite this, technological developments have substantially transformed dispensing practices. Nationally implemented innovations include electronic prescriptions, electronic medicines information systems, and more recently, comprehensive medication risk management systems supporting collaborative medication reviews.17., 19, 24., 26., 27., 28, 29 These technologies have contributed to a reduction in dispensing errors, particularly following the nationwide implementation of electronic prescriptions in 2017 and the introduction of the Medicines Verification System (MVS), maintained by the Finnish Medicines Verification Organization (FiMVO), in 2019.^29^

COVID-19 was classified as a generally hazardous communicable disease in Finland on 13 February 2020.^1^ Following the WHO pandemic declaration in March 2020, Finland entered a state of emergency, requiring community pharmacies to rapidly reorganize their services to meet increased demand while minimizing infection risks.30., 31. Addressing these challenges required close collaboration across the pharmaceutical supply chain.^3^

### 2.1 Aim of the study

Given the lack of research evidence on changes in digital and remote pharmacy services in Finland, we sought to determine which digital and remote services were offered by Finnish community pharmacies before and after the first wave of the COVID-19 pandemic. Because the Finnish pharmacy system is predominantly privately owned and pharmacy owners independently decide whether to introduce new operating models and services, we focused on the perspectives of pharmacy owners. The aim of this national cross-sectional study was to explore community pharmacy owners' readiness to adopt innovations and examine how this readiness was reflected in the availability of remote and digital services during the first wave of the COVID-19 pandemic in Finnish community pharmacies in 2020.

## 3 Methods

### 3.1 Study design and data collection

This study was conducted as a nationwide cross-sectional electronic survey using the Webropol data collection platform during October–November 2020. The survey collected information on electronic and remote services offered by community pharmacies and utilized by their customers in December 2019 (before the COVID-19 outbreak) and in October–November 2020 (approximately seven months after the onset of the pandemic). Thus, the reference period covered approximately 10 months.

An invitation to participate in the study, together with a link to the survey, was distributed by the personnel of the Association of Finnish Pharmacies (AFP) to all its members (*n* = 619), representing the owners of private community pharmacies in Finland. Only few pharmacy owners do not belong to the AFP, so the coverage of the study was almost complete in this respect. The pharmacy owners who responded provided information on behalf of their main pharmacy and possible branch pharmacies (i.e., on behalf of their pharmacy company). The researchers did not have access to the respondents' contact information. The survey instructions recommended that the pharmacy owner or, if unavailable, the owner's deputy complete the questionnaire.

### 3.2 Survey instrument

The survey instrument was developed by the researchers in collaboration with the Association of Finnish Pharmacies. The final survey instrument is available in Appendix B. It consisted of three sections: (1) questions concerning the characteristics of the responding community pharmacies and their owners; (2) questions specifically developed by the researchers to assess digital and remote services provided by Finnish community pharmacies before the COVID-19 outbreak (December 2019) and during the pandemic (October–November 2020); and (3) questions based on Rogers' Diffusion of Innovations theory concerning readiness to adopt new innovations (25, Appendix A).

We were unable to identify suitable validated survey items in the literature addressing the implementation of remote and digital services in community pharmacies. Therefore, we developed and pilot-tested new measures for face validity. These measures covered all digital and remote services known to be available in Finnish community pharmacies at the time of data collection (Table 1, Appendix B). All services included in the survey instrument were available in at least some Finnish community pharmacies in December 2019 (Table 1, Appendix B). Respondents were also given the opportunity to report any additional digital or remote services offered by their pharmacies.

**Table 1:** Digital and remote services provided by Finnish private community pharmacies in 2020 included in the survey instrument. The list included all known services that were available at least in some pharmacies. Respondents were also given the opportunity to report any additional digital or remote services offered by their pharmacies.

Electronic identification in connection with telephone transactions
Reservation of a prescription medicine using a smartphone application (e.g. Receptum's Mobile Prescription)
Electronic medication subscription service for healthcare organizations (e.g. Pharmadata's EasyMedi)
Medicine pick-up locker (the pharmacy will send a text message to the customer when the order is available for pickup from the locker)
Video call (prescription delivery)
Pharmacy's online service
Chat service
Medication review by phone
Doctor's remote appointment
Community pharmacist's remote medication counselling appointment for individual customers
Community pharmacist's remote appointment for organizations

The electronic subscription service for organizations is a system that enables authorized personnel to order medicines and other pharmacy products for nursing homes and home care units.32., 33. The video call service combines a smartphone application with a secure real-time consultation between a pharmacist and a customer. During the consultation, the pharmacist can provide counselling and remotely dispense medicines and other pharmacy products.^34^

The survey instrument primarily consisted of structured questions. A Likert-type scale was used in questions concerning the provision of electronic and remote services. Respondents were asked to report separately the availability, implementation status, and frequency of use of each service. For service availability and implementation status, the response options^1^, ^2^, ^3^, ^4^ were: “not planned,” “planned,” “implementation started,” and “operational” (Appendix B: Questions 12 and 14). For the use of electronic and remote services, the response options^1^, ^2^, ^3^, ^4^, ^5^ were: “service not available,” “no use,” “occasional use,” “weekly use,” and “daily use.” (Appendix B: Questions 13 and 15). The results of the responses regarding the availability of services and the extent of their adoption (“not planned”, “planned”, “setup started” and “operational”) made it possible to identify changes in service provision during the observation period.

Standard background questions routinely used by the Association of Finnish Pharmacies in previous surveys were incorporated into the questionnaire (Appendix B, Questions 1–11). These questions concerned pharmacy characteristics (e.g., annual prescription volume and location), pharmacy owner characteristics (e.g., age and years of experience), and pharmacy activities related to the implementation of new electronic and remote services during the study period. All other survey items, except the background questions and the statements assessing readiness to adopt innovations based on Rogers' theory,^25^ were developed by the research team.

The survey instrument was pre-tested for face validity by two private community pharmacy owners. Based on their feedback, minor revisions were made before the questionnaire was distributed to all private community pharmacy owners (Appendix B). Responses obtained during the pre-test were excluded from the final data analysis.

### 3.3 Theoretical framework

The study was guided by Rogers' Diffusion of Innovations theory as its theoretical framework.^25^ The survey instrument included a validated question assessing respondents' readiness to adopt innovations based on Rogers' theory.^25^ According to the theory, adopters can be categorized into five groups, as presented in Appendix A. In the Finnish pharmacy system, pharmacy owners independently decide which services are provided by their pharmacies, provided that these services promote the rational use of medicines. The introduction of new digital services, such as online pharmacy services, is voluntary. Consequently, pharmacies differ in their readiness to introduce new services and in their attitudes towards innovation and organizational change.

The question concerning innovation adoption behaviour “Which of the following options best describes your own attitude towards changes and new operating models?” consisted of the following 6 statements “I want to be involved in testing and development work immediately”; “I implement innovations more quickly than average and am happy to share my experiences with others”; “I do not implement innovations until their benefits have been demonstrated”; “I need time to consider changes and require support”; “I do not want changes, I am satisfied with the current situation and prefer established routines”; and “I cannot say” (Appendix B: Question 21). In addition, respondents were invited to share their experiences and opinions through open-ended questions (Appendix B).

### 3.4 Data collection and analysis

Respondents were instructed to submit one response per pharmacy. They were given 33 days to complete the survey. During the data collection period, three reminder emails were distributed by AFP? to encourage participation.

The response rate was calculated using the total number of AFP members as the denominator. No non-response analysis was conducted, and the characteristics of non-respondents were not examined. All responses were anonymous, and the researchers had no access to information that could identify either respondents or non-respondents. It is possible that non-respondents were generally less active in their roles as pharmacy owners; however, this assumption could not be verified.

For statistical analyses, respondents were classified into two groups according to their innovation adopter category: early adopters and late adopters (*n* = 170). The early-adopter group comprised innovators and early adopters, whereas the late-adopter group included the early majority, late majority, laggards, and respondents who selected “cannot say” (25, Appendix A). The classification of respondents into early and late adopters was performed after data collection.

Differences in service provision between the groups in December 2019 and October 2020 were assessed using the chi-square test of independence in Microsoft Excel.

Changes in the availability of medicine pick-up lockers and online pharmacy services between 2019 and 2020 were examined using McNemar's test for paired comparisons. Separate analyses were conducted for each service. Observations with missing values were excluded from the analyses, reducing the number of observations from 175 to 169 and 168, respectively.

Changes occurring during the observation period from December 2019 to October 2020 were analysed using the Wilcoxon signed-rank test. Separate analyses were conducted for the early-adopter and late-adopter groups. Observations with missing values were excluded from the analyses, reducing the number of observations from 175 to 155. McNemar's and Wilcoxon tests were performed using Jamovi software (version 2.7.33).

Multivariable analyses were conducted to explore associations between respondents' background characteristics and innovation adoption behaviour. The association between background characteristics and readiness to adopt innovations was examined using multivariable ordinal logistic regression. Logistic regression was selected because the characteristics of the data made it suitable for modelling the relationship between adoption behaviour and the explanatory variables.

The initial model included readiness to adopt innovations (five response categories) as the dependent variable and age, years of experience as a pharmacy owner, location, annual prescription volume, provision of dose-dispensing services, and the existence of a pharmacy strategy as explanatory variables. Potential confounding factors were assessed by sequentially removing variables from the model and examining changes in the estimated coefficients. Multivariable ordinal logistic regression analyses were performed using Jamovi software (version 2.7.33).

Information on new registrations of online pharmacy services by private pharmacies between 2011 and 2024 was obtained from publicly available statistics published by the Finnish Medicines Agency.^35^ The total number of notifications concerning online pharmacy services submitted to the regulatory authority during the same period was verified in March 2025.^36^ For the purpose of illustrating trends in annual online pharmacy registrations, pharmacy owners were classified into adopter categories based on the Diffusion of Innovations theory.^25^

### 3.5 Research ethics

The study was conducted in accordance with the Finnish Code of Conduct for Research Integrity and the national guidelines for responsible research conduct.^37^ Ethical pre-evaluation and approval was not required because the survey did not involve patients or medical interventions, collect personal data, health-related information, or other sensitive information.

Potential participants were informed about the purpose of the study and the handling of research data before completing the survey. Participation was voluntary, and submission of a completed questionnaire was regarded as providing informed consent. All responses were anonymous and contained no personal or health-related information. The researchers did not have access to respondents' contact details at any stage of the study.

The data were used exclusively for research purposes. Additional research material was obtained from publicly available statistics maintained by Finnish authorities. All study data were stored securely and protected from unauthorized access.

## 4 Results

### 4.1 Respondents and dichotomized adoption rate

Responses were received from 175 Finnish community pharmacies, yielding a response rate of 28%. The Association of Finnish Pharmacies had 619 member pharmacies at the time of data collection. Of the respondents, 170 (97%) answered the question concerning their readiness to adopt new innovations.

The distribution of respondents across innovation adopter categories was as follows: innovators 13% (*n* = 22), early adopters 30% (*n* = 51), early majority 40% (*n* = 67), late majority 15% (*n* = 25), laggards 0% (*n* = 0), and cannot say 3% (n = 5). The distribution was skewed towards earlier adoption categories. Based on annual prescription volume, larger pharmacies were overrepresented among respondents compared with the national distribution of community pharmacies.^38^

Early and late adopters were classified after data collection. Respondents were divided into two categories according to their self-assessed innovation adoption behaviour. Innovators and early adopters (43%, *n* = 73) were classified as early adopters, whereas respondents belonging to the early majority, late majority, laggards, and those selecting “cannot say” (57%, *n* = 96) were classified as late adopters (Appendix A). This dichotomization resulted in two groups of comparable size for subsequent analyses.

The multivariable ordinal logistic regression model provided evidence of factors associated with innovation adoption readiness. Potential confounding was assessed by sequentially removing variables from the model and evaluating changes in the estimated regression coefficients. Innovation adoption readiness was significantly higher among pharmacy owners aged under 50 years compared with those aged 50–59 years (*p* = 0.009) and those aged 60 years or older (*p* = 0.039). In addition, pharmacies with an annual prescription volume exceeding 100,000 prescriptions were more likely to demonstrate higher innovation adoption readiness than pharmacies dispensing fewer than 40,000 prescriptions annually (*p* = 0.016) (Table 2).

**Table 2:** Characteristics of respondents (*n* = 170) according to their self-assessed readiness to adopt new innovations (% of respondents) and the importance of these characteristics as a factor improving readiness to adopt determined using multivariate analysis.^25^

Variable	Early adopters n (%)	Late adopters n (%)	Total n (%)	Significance as adoption willingness enhancing factor
Age (years)	**n** **=** **73**	**n** **=** **96**	**n** **=** **169**	
<50	19 (26)	14 (15)	33 (19)	**Significant**
50–59	29 (40)	50 (52)	79 (45)	<50 vs. 50–59 ***p*** **=** **0.009**
60+	25 (34)	32 (33)	57 (33)	<50 vs. 60+ **p** **=** **0.039**
Years of experience as a pharmacy owner	**n** **=** **73**	***n*** **=** **97**	**n** **=** **170**	
<5	15 (21)	27 (28)	42 (25)	Not significant
5–10	22 (30)	30 (31)	52 (30)	Not significant
>10	36 (49)	40 (41)	76 (45)	Not significant
Annual prescription volume	***n*** **=** **68**	***n*** **=** **92**	***n*** **=** **160**	
<40,000	5 (7)	20 (22)	25 (16)	>100,000 vs. <40,000 **p** **=** **0.016**
40,000–60,000	10 (15)	18 (19)	28 (17)	40,000–60,000 vs. <40,000 *p* = 0.598
60,001–100,000	16 (24)	31 (34)	47 (29)	60,001–100,000 vs. <40,000 *p* = 0.101
>100,000	37 (54)	23 (25)	60 (38)	**Significant**
Location	***n*** **=** **71**	**n** **=** **97**	**n** **=** **168**	
Rural village/town	14 (20)	35 (36)	49 (29)	Not significant
Rural business center	5 (7)	7 (7)	12 (7)	Not significant
Suburb	7 (10)	11 (11)	18 (10)	Not significant
City center	19 (27)	16 (16)	35 (22)	Not significant
Shopping center or department store at the city center	26 (36)	28 (29)	54 (32)	Not significant
Automated dose dispensing	**n** **=** **73**	**n** **=** **97**	**n** **=** **170**	
Yes	65 (89)	79 (81)	144 (85)	Not significant
Written business strategy	***n*** **=** **72**	**n** **=** **96**	**n** **=** **168**	
Yes	42 (58)	45 (47)	87 (51)	Not significant

### 4.2 Service provision

All services included in the survey instrument were available in at least some Finnish community pharmacies in December 2019 (Table 1). Responses concerning service availability and stages of adoption (“not planned”, “planned”, “implementation initiated”, and “operational”) were used to assess changes during the observation period.

**Fig. 1:**
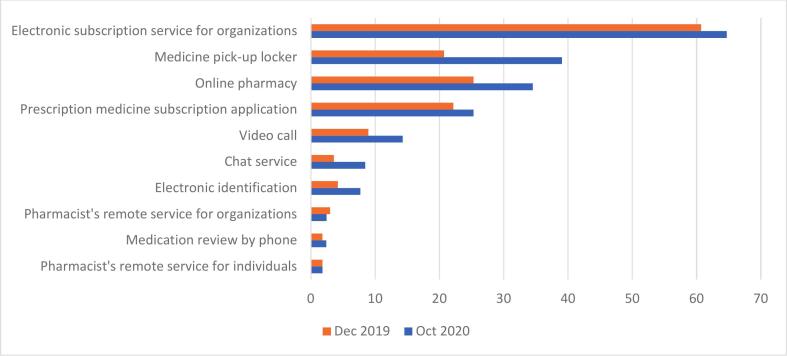
The prevalence of digital and remote services offered by Finnish community pharmacies in percentages in December 2019 (before the COVID-19 outbreak) and in October 2020 (7 months after the outbreak in March 2020) (*n* = 175).

Before the COVID-19 outbreak, the most commonly offered remote service among Finnish community pharmacies was an electronic subscription service for healthcare organizations, which was available in 61% of responding pharmacies in December 2019. This was followed by online pharmacy services (25%), medicine pick-up lockers (21%), prescription medicine reservation via a smartphone application (21%), and video consultation services for prescription medicine customers (9%) (Fig. 1).

The most notable change during the 10-month observation period from December 2019 to October 2020 was the increase in the number of pharmacies offering medicine pick-up lockers. The proportion of pharmacies providing this service increased from 21% in December 2019 to 39% in October 2020 (*p* = 0.001), representing an almost twofold increase in service provision. During the same period, the proportion of community pharmacies offering online pharmacy services increased from 25% to 34% (*p* = 0.001).

Electronic identification for telephone transactions, chat services, remote medication review services conducted by telephone, remote physician consultations, and pharmacists' remote medication counselling services were available only in a small number of pharmacies. In December 2019, each of these services was provided by fewer than 5% of the responding pharmacies (*n* = 175), and by October 2020, their availability remained below 9% among the same pharmacies (n = 175).

The remote and digital pharmacy services available during the early phase of the COVID-19 pandemic can be classified into two categories: (1) services related to the medicine delivery process (logistical services) and (2) services supporting appropriate medication use (care-oriented clinical pharmacy services). The remote pharmacy services that experienced the greatest growth in Finland following the onset of the pandemic in 2020 were those related to medicine delivery and logistics. In contrast, the provision of remote clinical pharmacy services remained limited. In 2019, services supporting rational pharmacotherapy were offered by fewer than 5% of the responding pharmacies, and no statistically significant changes were observed during the 10-month observation period.

Influence of Innovation Adoption Rate on the Introduction of Electronic and Remote Services.

**Fig. 2:**
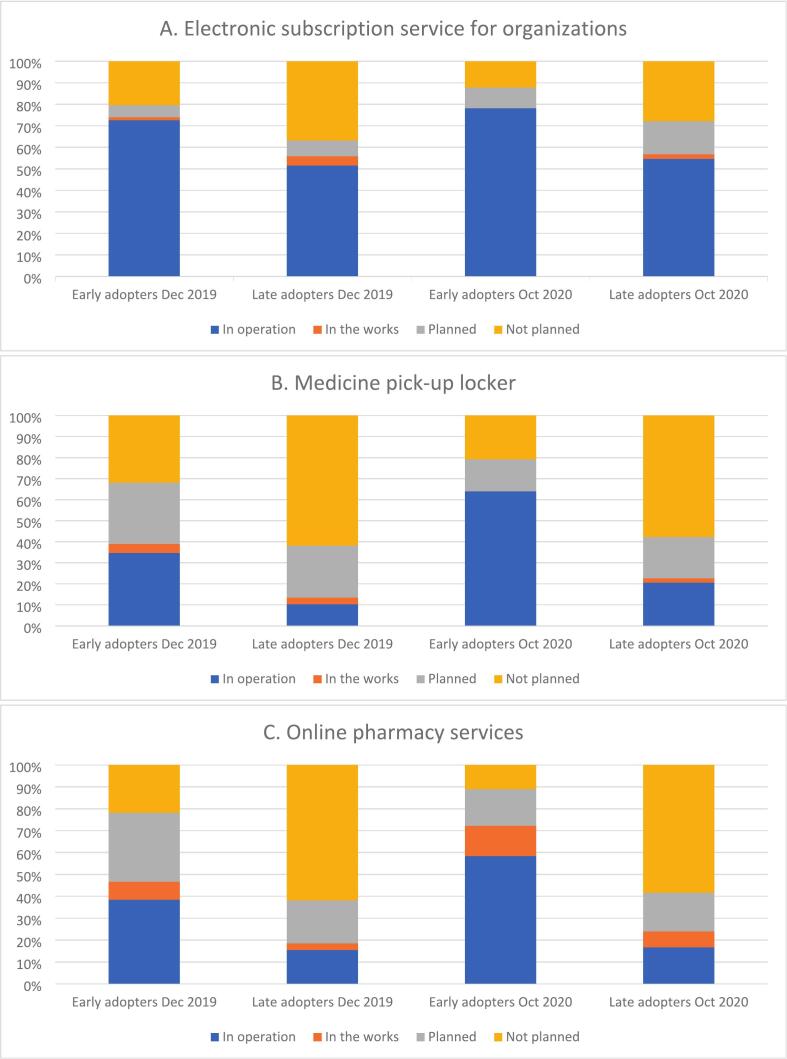
Availability of (A) electronic subscription services for healthcare organizations (*n* = 170), (B) medicine pick-up locker services (*n* = 169), and (C) online pharmacy services (*n* = 168) in Finnish community pharmacies in December 2019 and October 2020, according to innovation adoption category (% of responding pharmacies). Comparisons were made between early-adopter and late-adopter pharmacies and between the beginning and the end of the study period.

The differences in the number of remote services offered by early-adopter and late-adopter pharmacies in December 2019 and October 2020 are presented in Fig. 2. All five services were offered more frequently by pharmacies classified as early adopters than by those classified as late adopters at both time points. By October 2020, seven months after the COVID-19 outbreak in March 2020, the differences between the groups had become more pronounced (Table 3, Table 4). (See Fig. 2.)

**Table 3:** The five most common electronic and remote services offered by Finnish community pharmacies in December 2019 and October 2020, according to innovation adoption category (early adopters vs. late adopters). Differences between groups were analysed using the chi-square test of independence.

Remote service	Number of pharmacies where the service was available in December 2019		Significance of the difference between the groups “Early adopters” and “Late adopters” in December 2019 (*p* < 0.05)	Number of pharmacies where the service was available in October 2020		Significance of the difference between the groups “Early adopters” and “Late adopters” in October 2020 (p < 0.05)
	Early adopters % (n)	Late adopters % (n)		Early adopters % (n)	Late adopters % (n)	
Electronic subscription service for organizations	53 (73)	49 (95)	p < 0.05	57 (73)	53 (97)	*p* < 0.02
Medicine pick-up locker	25 (72)	10 (97)	p < 0.01	46 (72)	20 (97)	p < 0.01
Online pharmacy	28 (73)	15 (97)	p < 0.01	42 (72)	16 (96)	p < 0.01
Prescription medicine subscription application	19 (73)	18 (94)	No significant difference	27 (73)	16 (97)	p < 0.02
Video call	13 (72)	2 (96)	p < 0.01	16 (71)	8 (97)	p = 0.03

**Table 4:** Changes in the provision of electronic and remote services among early-adopter and late-adopter pharmacies between December 2019 and October 2020. Differences over time were assessed using the Wilcoxon signed-rank test. The analysis incorporated progression through all stages of service implementation (not planned, planned, implementation initiated, and operational).

Adopter type and service	n	Tied (n)	Changed (%)	Wilcoxon p
*Early adopters*				
Electronic identification in connection with telephone transactions	73	48	34.2	**0.002**
Prescription medicine subscription application	73	56	23.3	**<0.001**
Electronic medication subscription service for organizations	73	64	12.3	**0.007**
Medicine pick-up locker	71	48	32.4	**<0.001**
Video call	71	63	11.3	0.162
Online pharmacy	72	43	40.3	**<0.001**
Chat service	69	51	26.1	**0.001**
Medication review by phone	71	62	12.7	0.454
Pharmacist's remote medication counselling appointment for individual customers	70	63	10.0	0.073
Pharmacist's remote appointment for organizations	71	64	9.9	0.299

*Late adopters*				
Electronic identification in connection with telephone transactions	94	77	18.1	**0.023**
Prescription medicine subscription application	94	82	12.8	0.659
Electronic medication subscription service for organizations	95	81	14.7	0.062
Medicine pick-up locker	97	80	17.5	**0.001**
Video call	96	87	9.4	**0.040**
Online pharmacy	96	82	14.6	**0.008**
Chat service	95	81	14.7	0.488
Medication review by phone	95	89	6.3	0.374
Pharmacist's remote medication counselling appointment for individual customers	93	90	3.2	0.586
Pharmacist's remote appointment for organizations	93	91	2.2	0.346

The increase in the adoption of remote and electronic services was more pronounced among early-adopter pharmacies than among late-adopter pharmacies. The findings presented in Table 4 support the validity of the self-assessed innovation adoption measure, as pharmacies classified as early adopters exhibited more extensive implementation of new services during the study period. The statistically significant Wilcoxon signed-rank test results further indicate that service implementation progressed beyond what could be inferred solely from the comparison of service availability in December 2019 and October 2020.

### 4.3 Use of digital and remote services

**Fig. 3:**
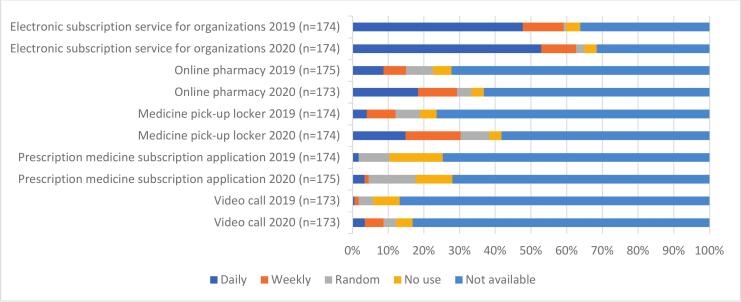
Reported frequency of use of the five most commonly used digital and remote services in Finnish community pharmacies in December 2019 and October 2020 (% of responding pharmacies).

The most widely used digital service was the electronic subscription service for healthcare organizations (Fig. 3). In December 2019, before the COVID-19 outbreak, the service was used daily by 48% of responding pharmacies (*n* = 174). By the end of the 10-month study period, daily use had increased to 53% of pharmacies (*n* = 175).

The greatest increase in service utilization was observed for medicine pick-up lockers. In December 2019, medicine pick-up lockers were used daily by 4% and weekly by 8% of pharmacies (*n* = 174). After 10 months, the corresponding proportions had increased to 15% for daily use and 15% for weekly use (*n* = 175).

During the same period, daily use of online pharmacy services increased from 9% (*n* = 173) to 18% (n = 174) of responding pharmacies, while weekly use increased from 6% to 11%.

Private customers visiting community pharmacies represented the most common user group of electronic and remote services among the responding pharmacies (*n* = 140). In contrast, the electronic subscription service was used predominantly by organizational customers (*n* = 121), including municipal home care services (36% of service users), private nursing homes (31%), and municipal nursing homes (29%).

### 4.4 Changes in the number of registered online pharmacy services during 2011–2024

**Fig. 4:**
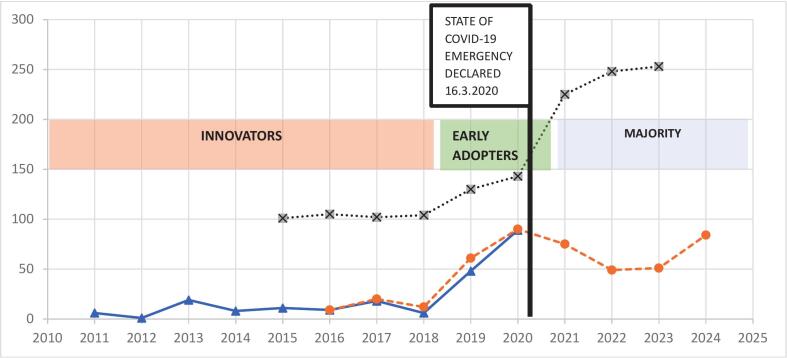
Annual number of new online pharmacy service registrations by Finnish community pharmacies in 2011–2020 (solid blue line), total number of online pharmacy service notifications submitted to the regulatory authority in 2016–2024 (dashed red line), and number of pharmacies providing one or more remote sales services (dotted black line), based on data from the Finnish Medicines Agency.35., 36., 39., 40. Online pharmacy founders were classified into adopter categories according to Rogers' Diffusion of Innovations theory using the annual number of online pharmacy registrations as the basis for classification.^25^ (For interpretation of the references to colour in this figure legend, the reader is referred to the web version of this article.)

Between 2011 and 2018, the Finnish Medicines Agency registered a maximum of 20 new community pharmacy online services annually, with a mean of 9.8 registrations per year (range 1–19) (35, Fig. 4). A marked increase occurred in 2019, when 61 new registrations were recorded, representing a more than fivefold increase compared with the previous year. Growth continued in 2020, when 90 new online pharmacy services were registered. This represented a 48% increase compared with 2019.

Fig. 4 presents the classification of online pharmacy founders according to the annual number of online pharmacy registrations.^25^ By the end of 2018, a total of 75 online pharmacies had been registered. Consequently, only 12% of Finnish community pharmacies (*n* = 619) had registered an online pharmacy before December 2019, when the growth in online pharmacy registrations accelerated substantially.

The Finnish Medicines Agency has modified its reporting practices regarding online pharmacy statistics since 2011, and data on new online pharmacy registrations are no longer available after 2020. Therefore, the figures presented for the period after 2020 include all notifications concerning online pharmacy services submitted to the regulatory authority.36., 39., 40.

Following the first wave of the COVID-19 pandemic, the increase in notifications stabilised. Nevertheless, the number of pharmacies providing online services continued to grow, as shown in Fig. 4. The readiness to adopt new innovations influenced the timing of the registration of online pharmacies and other digital services. In this context, registration of online pharmacy services reflects the actual adoption behaviour of pharmacy owners. The most rapid growth in new registrations occurred before the COVID-19 pandemic; however, widespread implementation of online services among community pharmacies took place after the pandemic had been declared. The differences observed between early and late adopters further highlight the importance of innovation readiness in the development and implementation of pharmacy services.

## 5 Discussion

Most digital and remote pharmacy services during the early COVID-19 pandemic were available in fewer than 10% of pharmacies. The most widespread service in Finnish community pharmacies was the electronic subscription service for organizations, whose availability and use increased moderately over the 10-month review period. Notably, private pharmacies had already widely adopted this service before the pandemic. In contrast, the pandemic clearly accelerated the adoption of remote services for private customers in 2020, with medicine pick-up lockers showing the greatest growth—their availability nearly doubled, reaching 39% (*n* = 169) of pharmacies by October 2020. Similar growth in locker services has also been observed in the Netherlands.^8^

A Dutch study during spring 2020 found that 44% of responding pharmacies (*n* = 215) conducted medication reviews, mainly by phone,^41^ while in Finland only 2% (*n* = 168) reported the same. This indicates that services supporting rational pharmacotherapy remain uncommon in Finland, even in remote form. International evidence shows that pharmacist-led medication reviews can reduce risks and improve adherence, especially among older adults.^42^ In Finland, automated dose dispensing has been the only widely implemented medication safety service for older patients with polypharmacy,^43^ suggesting that Finnish pharmacies should assume a stronger role in medication safety.^19^

Remote services related to medicine delivery had already begun increasing before 2020, driven by technological development, collaboration between pharmacies and system suppliers, and support from professional organizations,^21^ alongside established regulatory oversight.^24^ More broadly, telehealth adoption in community pharmacies has expanded rapidly across Western countries during COVID-19, improving continuity of care in both urban and rural areas.^41^, ^44^, ^45^ Before the pandemic, remote pharmacy services were mainly established in North America and Western Europe.^5^, ^6^, ^10^ The WHO predicted growing demand for digital medicine purchasing and consultation services, particularly for customers with limited mobility or long travel distances.^46^ Remote services also offer discreet access to care^10^, ^43^ and have often been developed to address gaps in pharmacy availability in remote areas.^10^, ^47^

The spread of remote pharmaceutical services ultimately depends on user demand.^47^ General digitalisation has enabled the rise of online pharmacies,^48^ which have been authorized in Finland since 2011. Finnish pharmacy owners appear relatively quick adopters of innovations in line with Rogers' diffusion of innovations theory.17., 25 By 2020, all EU countries had improved digital performance,^49^ and Finland ranks among global leaders in digitalisation, with highly active internet users.^49^ This supports the adoption of remote pharmacy services nationwide, not only in remote areas, since pharmacies are evenly distributed by regulation.

From a diffusion perspective, first adopters of online pharmacy services can be classified as innovators (Fig. 4). In this study, 13% of respondents identified as eager to participate in testing and development, aligning with this category. Pharmacies that established online operations between 2011 and 2018 had to address technical, logistical, and legal challenges independently. By 2018, 75 pharmacies (12%) had registered online services (*n* = 619 benchmark). The COVID-19 pandemic further accelerated adoption, as pharmacies sought to reduce physical contact, reflected in a sharp increase in registrations in 2020.

### 5.1 Strengths and limitations

We used a survey instrument consisting primarily of structured questions, supplemented with a small number of open-ended questions, to collect the study data. A four-point Likert scale was applied to questions concerning the planning, implementation, and provision of digital and remote services, enabling the use of quantitative statistical analyses.

As with all survey-based research, the findings are subject to potential sources of bias. Data were collected using a self-administered questionnaire because no national registry data are available on the provision of digital and remote pharmacy services. The accuracy of the responses may have been influenced by whether the questionnaire was completed by a pharmacy owner or a deputy. Although respondents were instructed that the pharmacy owner or deputy could complete the survey, information on which respondents were deputies was not collected.

The survey was conducted approximately 10 months after the beginning of the study period. Consequently, respondents were required to report retrospectively on services provided before and during the early stages of the COVID-19 pandemic. This may have introduced recall bias. In addition, self-reported surveys are susceptible to social desirability bias, whereby respondents may overestimate or overreport positive behaviours and activities.

Compared with the distribution of adopter categories proposed by Rogers' Diffusion of Innovations theory, our study identified a considerably higher proportion of innovators among respondents (13.0% vs. 2.5%). This finding suggests that more innovation-oriented pharmacy owners may have been more willing to participate in the survey than those less interested in adopting new services. Consequently, the results may present a more favourable picture of innovativeness among Finnish community pharmacy owners than exists in the overall population.

The use of a voluntary self-assessment questionnaire also introduces the possibility of non-response bias, particularly when response rates are relatively low.^50^ In the present study, the response rate was 28%. Respondents classified as early adopters appeared to be overrepresented, further indicating the potential presence of non-response bias. Therefore, the findings should be interpreted with caution and may not be fully generalizable to all Finnish community pharmacy owners.

Despite these limitations, the study provides valuable information on the adoption and provision of digital and remote pharmacy services in Finland during the COVID-19 pandemic. The findings may assist community pharmacies in further developing remote service models and supporting digital transformation. Further research is needed to examine how the market for digital pharmacy services has evolved following the pandemic and to explore effective strategies for developing, implementing, and sustaining digital clinical pharmacy services.

## 6 Conclusions

The COVID-19 pandemic accelerated the adoption and implementation of digital and remote services in Finnish community pharmacies. This development was facilitated by the availability of technological solutions and by pharmacy owners' generally positive attitudes towards innovation. The differences observed between early and late adopters highlight the important role of innovation readiness in the development and uptake of new pharmacy services.

Community pharmacies responded rapidly to changes in the operating environment. The swift expansion of online pharmacy services, medicine pick-up lockers, and telepharmacy solutions demonstrates that pharmacy services are no longer confined to physical premises or traditional opening hours.

Healthcare organizations also appear to be well positioned to collaborate electronically with community pharmacies. The rapid growth of online pharmacy services and increasing customer interest in digital pharmacy channels suggest considerable potential for the further expansion of e-commerce and other digitally enabled pharmacy services in the future.

## Declaration of generative AI and AI-assisted technologies in the manuscript preparation process

During the preparation of this work, the authors used MS Copilot for language check. The authors reviewed and edited the output as needed and take full responsibility for the content of the published article.

## CRediT authorship contribution statement

**Paula Timonen:** Writing – review & editing, Writing – original draft, Visualization, Validation, Resources, Project administration, Investigation, Formal analysis, Data curation, Conceptualization. **Katja Ojanperä:** Writing – review & editing, Validation, Methodology. **Eeva Savela:** Writing – review & editing, Validation, Supervision, Conceptualization. **Marja Airaksinen:** Writing – review & editing, Validation, Supervision, Methodology, Conceptualization.

## Funding

This research did not receive any specific grant from funding agencies in the public, commercial, or not-for-profit sectors.

## Declaration of competing interest

The authors declare that they have no known competing financial interests or personal relationships that could have appeared to influence the work reported in this paper.

## Figures and Tables

**Table:** 

Type of adopters	Characteristics
Innovators	Social, in the closest contact to scientific sources, interaction with other innovators, financial liquidity, the highest social status and willing to take risk.
Early adopters	More socially forward than late adopters, have the highest degree of opinion leadership among the adopter categories, a higher social status, financial liquidity, advanced education.
Early majority	Adopt an innovation after a varying degree of time that is significantly longer than in the case of innovators and early adopters, above average social status, contact with early adopters and seldom hold positions of opinion leadership in a system.
Late majority	Approach an innovation with a high degree of scepticism and after the majority of society has adopted the innovation, have below average social status and little financial liquidity.
Laggards	Typically have an aversion to change-agents, tend to be focused on “traditions”, have lowest social status and lowest financial liquidity, are oldest among adopters, and are in contact with only family and close friends.

## Appendix A

The classification of the types of adopters according to the theory of diffusion of innovations by Rogers.^23^ In this study the respondents were divided into two groups, early and late adopters. The group “Early adopters” included innovators and early adopters, and the group “Late adopters” included early majority, late majority, laggards and those who “cannot say”.^23^

## Appendix B

The questionnaire of the electronic survey.

1. Age of pharmacist at the time of response (answer options: < 50 years, 50–59 years, > 60 years)

2. Years of experience as a pharmacist (total length of pharmacist career)

a. Less than 5 years

b. 5–10 years

c. More than 10 years

3. Geographical location of the pharmacy

a. Uusimaa

b. Southwest Finland

c. Satakunta

d. Kanta-Häme

e. Pirkanmaa

f. Päijät-Häme

g. Kymenlaakso

h. South Karelia

i. South Savo

j. North Savo

k. North Karelia

l. Central Finland

m. South Ostrobothnia

n. Ostrobothnia

o. Central Ostrobothnia

p. North Ostrobothnia

r. Lapland

s. Åland

4. Local location of the pharmacy

a. Rural settlement

b. City, city centre area (so-called foothills)

c. City, other district/suburb

d. Shopping centre

5. Pharmacy prescriptions (main, branch and network pharmacies total) in 2019.

a. Under 40,00

b. 40,000 - 60,000

c. 60,001 - 100,000

d. Over 100,000

6. Pharmacy turnover

a. Under €1,700,000

b. €1,700,000 – €2,499,999

c. €2,500,000 – €3,399,999

d. €3,400,000 – €4,999,999

e. €5,000,000 or more

7. In addition to the main pharmacy, the pharmacy's operations include (you can select several)

a. Branch pharmacy

b. Service point

c. Membership in a marketing chain

d. Limited liability company

e. Online pharmacy

f. Health point

g. Electronic drug dispensing service

h. Other paid services

i. None of the above

8. Time since the online pharmacy was established (answer options: not established, < 1 year, 1–3 years, 3–6 years, > 6 years)

9. Time since the electronic drug dispensing service was started (answer options: not established, < 1 year, 1–3 years, 3–6 years, 6–10 years, > 10 years)

10. Does the pharmacy have a defined (written) operating strategy that it operates according to?

a. Yes

b. No

11. Has the operating strategy been reviewed with the staff?

a. Yes

b. No

12. Status of pharmacy electronic and remote services in December 2019 (answer options: not planned, planned, setup started, operational)

a. Electronic identification in connection with telephone transactions

b. Booking a prescription medication using a smartphone application (e.g. Receptum's Mobile Prescription)

c. Electronic medication subscription service for healthcare organizations (e.g. Pharmadata's EasyMedi)

d. Medicine pick-up locker (text message to the customer when the order is available for pickup)

e. Video call (prescription delivery)

f. Online pharmacy

g. Chat service

h. Medication review by phone

i. Doctor's remote appointment

j. Pharmacist's remote medication counselling appointment for individual customers

k. Pharmacist's remote appointment for organizations

l. Other electronic service or operating model, which one?

13. Use of electronic and remote services in December 2019 (answer options: service not available, no use, occasional use, weekly use, daily use)

a. Electronic identification in connection with telephone transactions

b. Booking a prescription using a smartphone application (e.g. Receptum's Mobile Prescription)

c. Electronic medication subscription service for healthcare organizations (e.g. Pharmadata's EasyMedi)

d. Medicine pick-up locker (text message to the customer when the order is available for pickup)

e. Video call (prescription delivery)

f. Online pharmacy

g. Chat service

h. Medication review by phone

i. Doctor's remote appointment

j. Pharmacist's remote medication counselling appointment for individual customers

k. Pharmacist's remote appointment for organizations

l. Other electronic service or operating model, which one?

14. Status of the pharmacy's electronic and remote services at the time of the response (answer options: not planned, planned, setup started, operational)

a. Electronic identification in connection with telephone transactions

b. Booking a prescription drug using a smartphone application (e.g. Receptum's Mobile Prescription)

c. Electronic medication subscription service for healthcare organizations (e.g. Pharmadata's EasyMedi)

d. Medicine pick-up locker (text message to the customer when the order is available for pickup).

e. Video call (prescription delivery)

f. Online pharmacy

g. Chat service

h. Medication review by phone

i. Doctor's remote appointment

j. Pharmacist's remote medication counselling appointment for individual customers

k. Pharmacist's remote appointment for organizations

l. Other electronic service or operating model, which one?

15. Use of electronic and remote services at the time of the response (answer options: service not available, no use, occasional use, weekly use, daily use)

a. Electronic identification in connection with telephone transactions.

b. Booking a prescription medication using a smartphone application (e.g. Receptum's Mobile Prescription

c. Electronic medication subscription service for healthcare organizations (e.g. Pharmadata's EasyMedi)

d. Medicine pick-up locker (text message to the customer when the order is available for pickup)

e. Video call (prescription delivery)

f. Online pharmacy

g. Chat service

h. Medication review by phone

i. Doctor's remote appointment

j. Pharmacist's remote medication counselling appointment for individual customers

k. Pharmacist's remote appointment for organizations

l. Other electronic service or operating model, which?

16. The most significant customer group for electronic and remote services at the time of the response (answer options: private individual customers, municipal home care, private home care, municipal nursing homes, private nursing homes, other organizations)

a. Electronic identification in connection with telephone transactions

b. Booking a prescription medication using a smartphone application (e.g. Receptum's Mobile Prescription)

c. Electronic medication subscription service for healthcare organizations (e.g. Pharmadata's EasyMedi)

d. Medicine pick-up locker (text message to the customer when the order is available for pickup)

e. Video call (prescription delivery)

f. Online pharmacy

g. Chat service

h. Medication review by phone

i. Doctor's remote appointment

j. Pharmacist's remote medication counselling appointment for individual customers

k. Pharmacist's remote appointment for organizations l. Other electronic service or operating model, which one?

17. Most common feedback from customers about electronic and remote services (answer options: negative, positive, I don't know)

a. Electronic identification when making telephone transactions

b. Booking a prescription using a smartphone application (e.g. Receptum's Mobile Prescription)

c. Electronic medication subscription service for healthcare organizations (e.g. Pharmadata's EasyMedi)

d. Medicine pick-up locker (text message to the customer when the order is available for pickup)

e. Video call (prescription delivery)

f. Online pharmacy

g. Chat service

h. Medication review by phone

i. Doctor's remote appointment

j. Pharmacist's remote medication counselling appointment for individual customers

k. Pharmacist's remote appointment for organizations

l. Other electronic service or operating model, which one?

18. The impact of electronic and remote services on the pharmacy's results at the time of response (Answer options: poor (costs are bigger than profit), moderate (just pays for itself), good (the activity produces profit), I can't say)

a. Electronic identification in connection with telephone transactions

b. Booking a prescription using a smartphone application (e.g. Receptum's Mobile Prescription)

c. Electronic medication subscription service for healthcare organizations (e.g. Pharmadata's EasyMedi)

d. Medicine pick-up locker (text message to the customer when the order is available for pickup)

e. Video call (prescription delivery)

f. Online pharmacy

g. Chat service

h. Medication review by phone

i. Doctor's remote appointment

j. Pharmacist's remote medication counselling appointment for individual customers

k. Pharmacist's remote appointment for organizations

l. Other electronic service or operating model, which one?

19. Has the pharmacy staff received training on electronic and remote services?

a. Internal pharmacy training

b. External pharmacy training

c. No training

20. What is the attitude of your pharmacy staff towards electronic and remote services?

a. Negative, inhibiting

b. Neutral or I can't say

c. Positive, enthusiastic

d. Attitudes vary greatly between individual customers

21. Which of the following options best describes your own attitude towards changes and new operating models?

a. I want to be involved in testing and development work immediately

b. I will implement innovations faster than average and I will be happy to tell others about them

c. I will not implement innovations until the benefits have been demonstrated

d. I need time to think and support

e. I do not want changes, I am satisfied with the current situation and like routines

f. I can't say

22. Open field for sharing experiences and opinions
